# An Evaluation of Serum IgE and Th2-Associated Interleukins in Children With Uncomplicated and Complicated Appendicitis

**DOI:** 10.3389/fped.2022.884138

**Published:** 2022-05-02

**Authors:** Johanna Gudjonsdottir, Bodil Roth, Gustav Lovén, Bodil Ohlsson, Lars Hagander, Martin Salö

**Affiliations:** ^1^Department of Clinical Sciences, Pediatrics, Lund University, Lund, Sweden; ^2^Department of Surgery, Skåne University Hospital, Malmö, Sweden; ^3^Department of Internal Medicine, Skåne University Hospital, Malmö, Sweden; ^4^Department of Clinical Sciences, Lund University, Malmö, Sweden; ^5^Department of Pediatric Surgery, Skåne University Hospital, Lund, Sweden

**Keywords:** IgE, Th2, interleukins, children, appendicitis

## Abstract

**Background:**

The pathogenesis of appendicitis is not understood completely and establishing a correct diagnosis can be clinically challenging. Previous investigations have shown an association between a T helper cell (Th)2-mediated inflammatory response, for example immunoglobulin E (IgE)-mediated allergy, and a decreased risk of complicated appendicitis. The present study aimed to evaluate differences in serum concentrations of IgE and Th2-associated interleukins (IL) in children with uncomplicated and complicated appendicitis.

**Method:**

A prospective study including children <15 years with appendicitis. Blood samples were collected preoperatively at the time of clinical assessment at the Pediatric Emergency Department and analyzed for concentrations of serum total IgE and IL-4, IL-9, and IL-13. Associations with complicated appendicitis were evaluated through logistic regression adjusting for age, appendicolith, and symptom duration.

**Results:**

138 children with confirmed appendicitis were included. The median age was 10 (IQR 8–12) years, 87 (63%) were boys and 58 (42%) had complicated appendicitis. Children with complicated appendicitis had significantly higher concentrations of IL-9 and IL-13 compared to children with uncomplicated appendicitis. In the univariate logistic regression, high concentrations of IL-13 were associated with an increased risk of complicated appendicitis [OR 1.02 (95% CI 1.01–1.04) *p* = 0.005], which remained in the multivariate analysis [aOR 1.02 (95% CI 1.01–1.04), *p* = 0.01]. Serum concentrations of IgE, IL-4, and IL-9 did not significantly affect the risk of complicated appendicitis.

**Conclusion:**

High levels of IL-13 seem to be associated with an increased risk of complicated appendicitis. This is incongruent with the hypothesis of an Th1/Th17-driven inflammation in this type of appendicitis.

## Introduction

Appendicitis is the most common non-traumatic abdominal emergency in children, and appendectomy is the most performed acute surgical procedure undertaken worldwide (1). The lifetime risk of appendicitis is 8.6% for men and 6.7% for women, and the incidence peaks during the second decade of life (2). Even though appendicitis is a very common condition, the rates of initial misdiagnoses are high, especially for younger children and girls (3, 4).

Appendicitis has long been considered a continuously progressive disease, inevitably resulting in perforation if left untreated. These beliefs have resulted in a general attitude promoting early surgery and an acceptance of high rates of negative appendectomies (5). During the last decades, however, it has become evident that not all cases of appendicitis progress to perforation. Rather, a substantial proportion of cases resolve spontaneously without any treatment (6–9). This has led to a paradigm shift in the perception of the pathophysiology of appendicitis, where uncomplicated appendicitis is less seen as a precursor of complicated appendicitis, but rather that uncomplicated and complicated appendicitis may constitute two different entities with unique pathogenesis and pathological aggressiveness. To differentiate clinically between the two different types of appendicitis can be challenging but is important to determine the proper evaluation and treatment strategies for these patients. It has been advocated that the management of patients with suspected appendicitis should be focused on diagnosing those with complicated disease for prompt surgical treatment (5), while children with uncomplicated appendicitis do not have to be rushed to the operating room or perhaps they will not require surgery at all (10).

Both epidemiological (11–13) and clinical (14) studies have suggested that a person's immune response propels the inflammation toward either an uncomplicated or a complicated disease course, where complicated appendicitis is associated with a T helper (Th) 1/Th17-dependent immune response and uncomplicated appendicitis is associated with a Th2 dependent immune response. We have reported previously that children with the Th2-associated condition IgE-mediated allergy have a substantially lower risk of developing complicated appendicitis, compared to non-allergic children (15, 16). However, the causal pathway, i.e., which immunological mechanisms are responsible for protection, remains to be investigated.

The aim of the present study was to evaluate whether concentrations of IgE and the Th2-associated cytokines interleukin (IL)-4, IL-9 and IL-13 in serum are associated with the risk of complicated appendicitis in children. We hypothesized that higher concentrations of these biomarkers would be associated with a decreased risk of complicated appendicitis.

## Materials and Methods

### Study Design

A prospective cohort study was performed during three consecutive years, from 9 December 2017 to 16 February 2021. Children were included at the Pediatric Emergency Department of a tertiary pediatric surgery center with an uptake area of 350,000 inhabitants for general surgical emergencies. The study was approved by the regional ethics board (Regionala Etikprövningsmyndigheten, Lund, Sweden, DNR 2013/614) and the hospital review board (Skåne University Hospital, Lund, Sweden). All included subjects agreed to participation through parental informed and written consent.

The main objective was to evaluate the association of different levels of IgE and Th2-associated interleukins with the risk of complicated appendicitis in a cohort of children with appendicitis. We also included a group of children with non-appendicitis acute abdominal pain, as sensitivity analysis adding robustness to the analyses and interpretation of levels of the biomarkers.

### Inclusion and Exclusion Criteria

All children (≤ 15 years of age) presenting to a pediatric surgeon with suspected appendicitis were eligible for inclusion in the study, regardless of the final diagnosis. Exclusion criteria were previous episodes of suspected appendicitis, severe chronic illness, or ongoing treatment with anti-inflammatory drugs (including inhaled corticosteroids).

### Data Collection

Data were collected prospectively at the Pediatric Emergency Department. The pediatric surgeon who examined the patients registered information on medical history including current symptom duration, findings on clinical examination, and results of standard laboratory tests in a study registry. Study blood samples were collected only if blood tests were also medically indicated as part of the clinical assessment at the Pediatric Emergency Department, in order not to expose the subjects to unnecessary discomfort. Medical records were reviewed to obtain information on the patient's allergy status at the time of inclusion and presence of an appendicolith (on radiology or perioperatively), and to determine the patient's final diagnoses and the results from histopathological examination.

### Primary Outcome, Exposure, and Independent Variables

Primary outcome was complicated appendicitis. The diagnosis was based on intraoperative findings and histopathological examination of removed appendices. Uncomplicated appendicitis was defined as phlegmonous appendicitis, with histopathological diagnosis criteria of infiltration of neutrophil granulocytes in the muscularis propria layer. Complicated appendicitis was defined as a gangrenous or perforated appendicitis, as well as an appendicular abscess. Gangrenous appendicitis was defined as an inflamed appendix with necrotic (gray or black) discoloration with histological signs of substantial tissue necrosis and absence of clinical signs of perforation. Perforated appendicitis was defined as a visual hole in a gangrenous appendicitis or the finding of an appendicolith or free pus or intestinal content in the abdominal cavity. The diagnosis of an appendiceal abscess was based on intraoperative or radiological findings.

Primary exposures were concentrations of serum total IgE and IL-4, IL-9, and IL-13.

Independent variables were age, sex, symptom duration, season, IgE-mediated allergy, and presence of an appendicolith. Symptom duration was measured from reported onset of symptoms to the visit at the Pediatric Emergency Department. Season was categorized as spring (March–May), summer (June–August), autumn (September–November) and winter (December–February). Allergy status was based on patient or parental reports and retrieved from medical charts. Appendicoliths were detected either intraoperatively or through preoperative ultrasonographic or computed tomographic studies.

### Analysis of IgE and Interleukins

Blood samples were collected at the Pediatric Emergency Department and sent to the Department of Clinical Chemistry. The test tubes containing blood were left standing for 30 min before centrifugation at 2000G. Serum was then allocated to one to three test tubes (depending on the amount of serum available) containing 0.5 mL and frozen to−80°C. The frozen serum samples were stored at the regional biobank until analyzed.

Human serum IgE was analyzed using a sandwich enzyme-linked immunosorbent assay (ELISA) kit (ab195216, Abcam, Netherlands) according to the manufacturer's manual. Standard (0, 0.11, 0.18, 0.26, 0.40, 0.59, 0.89 and 1.33 ng/mL) serum samples diluted 1:800 (50 μL/well in duplicate) and antibody cocktail (50 μL/well) were added. After 1 h's incubation at room temperature on a shaker, the plates were washed and tetramethylbenzidine (TMB) substrate 100 μL/well was added. The reaction was stopped after a 10-min incubation in darkness and the absorbance was measured at 450 nm. Intra-and inter assay coefficients of variations (CV) were 4.0% (*n* = 4) and 3.5% (*n* = 3), respectively, and average recovery was108% (range 98.4–118 %).

The Mesoscale Discovery^®^ (MSD, Maryland, USA) U-PLEX^®^ multiplex assay biomarker group (K15067L-2, MSD) was used to perform the selected analyses of IL-4, IL-9 and IL-13 in serum by electro-chemiluminescence detection. Biotinylated IL-4, IL-9 and IL-13 capture antibodies, 50 μL/well, were added to the U-PLEX™ multiplex SECTOR^®^ plate and incubated overnight at 4°C on a shaker. Calibrators 50 μL/well and serum (diluted 1:2) 50 μL/well were added after the plates had been washed three times with MSD wash buffer. A 1-h incubation at room temperature was followed by a new washing procedure and a SULFO-TAG™ detection antibody, 50 μL/well, was added. After a second 1-h incubation and a washing procedure, 150 μL MSD GOLD™ read buffer in each well was added and the plates were read on an MSD instrument. The intensity of emitted light is proportional to the amount of IL-4, IL-9 and IL-13 in the wells.

### Statistics

All statistical analyses were performed using IBM SPSS for Macintosh, version 25.0 (Armonk, NY: IBM Corp). Continuous non-normal distributed variables were reported as median (IQR), with differences between the two groups assessed using the Mann-Whitney U-test and three groups using the Kruskal-Wallis test with a *post-hoc* Dunn-Bonferroni test. Dichotomous variables were presented as frequencies and percentages, with differences between groups assessed using the Chi-squared test. Associations of independent variables and the primary outcome were assessed through logistic regression and presented as odds ratios (ORs) with 95% CIs. Variables with statistically significant associations to the primary outcome on univariate logistic regressions were included in the multivariable logistic regression model. Before logistic regression, the variable of total serum IgE- concentrations was dichotomized and the patients allocated to either above or below maximum reference intervals according to age-specific cut-offs (Supplementary Table 1).

## Results

**Figure 1:**
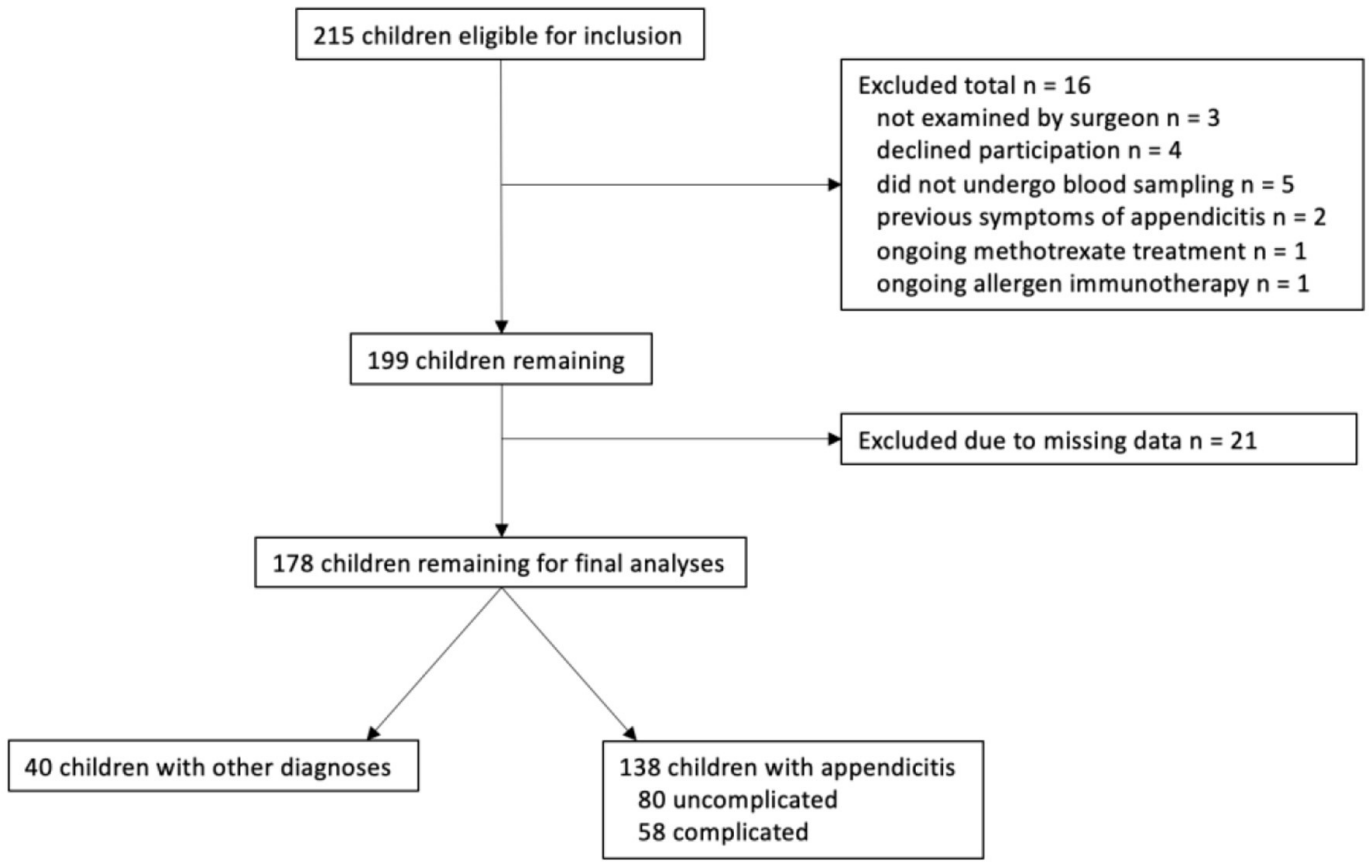
The inclusion and exclusion of 215 children with suspected appendicitis.

A total of 215 children were initially eligible for inclusion. Sixteen were excluded due to exclusion criteria and another 21 were excluded as a result of missing data (Figure 1). Therefore, 178 children remained for further analyses. Of these 138 (78%) had appendicitis; 80 (45%) had uncomplicated appendicitis and 58 (33%) had complicated appendicitis. Of the children with complicated appendicitis, 23 had gangrenous appendicitis, 31 had perforated appendicitis and four had an appendicular abscess. The median age of the children with appendicitis was 10 (IQR 8-12) years, and 87 (63%) were boys (Table 1). The control group consisted of the remaining 40 children with abdominal pain due to other diagnoses (Supplementary Table 2).

**Table 1:** Demographics of 138 children with uncomplicated and complicated appendicitis.

	**Uncomplicated** **appendicitis** ***n* = 80**	**Complicated** **appendicitis** ***n* = 58**	***p*-value**
Age (years)	11 (9–13)	9 (7–12)	**0.004**
Sex (male)	49 (61)	38 (66)	0.608
Allergy	15 (19)	5 (9)	0.095
**Symptom duration**
0–24 h	42 (53)	18 (31)	0.054
24–48 h	27 (34)	23 (40)	
48–96 h	10 (13)	14 (24)	
>96 h	0 (0)	2 (3)	
**Season**
Spring	16 (20)	17 (29)	0.213
Summer	14 (18)	15 (26)	
Autumn	20 (25)	12 (21)	
Winter	30 (38)	14 (24)	

*Values presented as median (IQR) and as absolute number and percentage of patients; n (%). Group differences were assessed through Mann-Whitney U test for continuous data (age) and with Chi-squared test for categorical data (sex, allergy status, symptom duration and season). Symptom duration n = 79 and 57. The bold values indicate the p-value below 0.05*.

**Figure 2:**
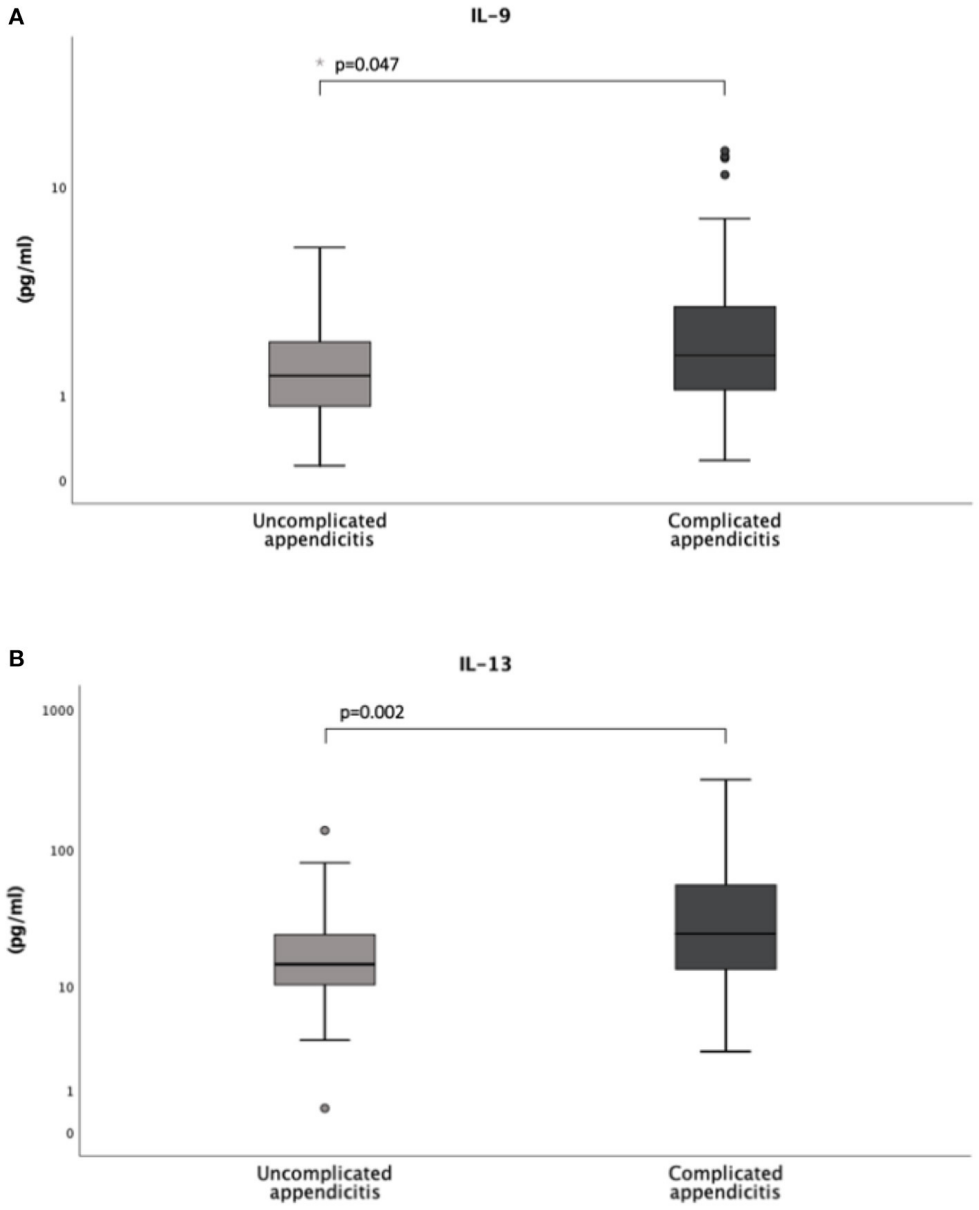
Levels of interleukin IL-9 **(A)** and IL-13 **(B)** in a cohort of 138 children with uncomplicated and complicated appendicitis. Both the circles and the asterisk (*) in the box plots represent outliers.

Concentrations of IL-9 and IL-13 were significantly higher in the children with complicated appendicitis [1.8 (IQR 1.1-3.2) pg/mL and 24.6 (IQR 12.9–58.5) pg/mL] compared to the children with uncomplicated appendicitis [1.4 (IQR 0.8–2.1) pg/mL and 14.6 (IQR 10.2–24.3) pg/mL, *p* = 0.047 and 0.002, respectively]. There were no significant differences in the concentrations of IL-4 and IgE (Table 2, Figure 2). Further analyses with comparison of the different biomarkers between the two appendicitis groups and the non-appendicitis group can be found in Supplementary Table 3, Supplementary Figure 1.

**Table 2:** Levels of interleukins and IgE in 138 children with uncomplicated and complicated appendicitis.

	**Uncomplicated** **appendicitis** ***n* = 80**	**Complicated** **appendicitis** ***n* = 58**	***p*-value**
Serum IgE (ng/mL)	160.8 (72.6–530.5)	127.2 (64.0–404.2)	0.159
Serum IL-4 (pg/mL)	0.3 (0.2–0.6)	0.4 (0.2–0.7)	0.896
Serum IL-9 (pg/mL)	1.4 (0.8–2.1)	1.8 (1.1–3.2)	**0.047**
Serum IL-13 (pg/mL)	14.6 (10.2–24.3)	24.6 (12.9–58.5)	**0.002**

*Values presented as median (IQR), group differences assessed through Mann-Whitney U test. S-IL-4 n = 72 and 50, since some values were unmeasurable. S-IL-9 n = 78 and 55. S-IL-13 n = 78 and 57. The bold values indicate the p-value below 0.05*.

In the univariate logistic regression analysis, higher concentrations of IL-13 were significantly associated with an increased risk of complicated appendicitis (OR 1.02 (95% CI 1.01–1.04), *p* = 0.005). This association remained after adjustment for age, symptom duration and presence of an appendicolith [aOR 1.02 (95% CI 1.01–1.04), *p* = 0.011] (**Table 4**). IgE, IL-4, and IL-9 concentrations did not affect the risk of complicated appendicitis in the crude (Table 3) or the adjusted analysis (Table 4): IgE aOR 0.53 (95% CI 0.329–1.181, *p* = 0.12), IL-4 aOR 1.08 (95% CI 0.59–1.99, *p* = 0.81), and IL-9 aOR 1.05 (95% CI 0.93–1.19, *p* = 0.46).

**Table 3:** Unadjusted independent variables of complicated appendicitis in 138 children with appendicitis.

	**Uncomplicated appendicitis** **(*n* = 80)**	**Complicated appendicitis** **(*n* = 58)**	**OR (95% CI)**	***p*-value**
Age (years)	11 (9–13)	9 (7–12)	0.84 (0.75–0.94)	**0.003**
Sex (male)	49 (61%)	38 (66%)	1.20 (0.60–2.43)	0.608
Allergy	15 (19%)	5 (9%)	0.41 (0.14–1.20)	0.103
**Symptom duration**				
0–24 h	42 (53%)	18 (31%)	Ref	Ref
24–48 h	27 (34%)	23 (40%)	1.99 (0.91–4.35)	0.086
48–96 h	10 (13%)	14 (24%)	3.27 (1.22–8.71)	**0.018**
>96 h	0 (0%)	2 (3%)	N/A	N/A
**Season**				
Spring	16 (20%)	17 (29%)	Ref	
Summer	14 (18%)	15 (26%)	(0.37–2.74)	0.987
Autumn	20 (25%)	12 (21%)	0.57 (0.21–1.52)	0.25
Winter	30 (38%)	14 (24%)	0.44 (0.17–1.12)	0.084
Appendicolith	13 (16%)	21 (36%)	2.86 (1.28–6.41)	**0.011**
Serum IgE elevated^*^	39 (49%)	21 (34%)	0.60 (0.30–1.19)	0.144
Serum IL-4 (pg/mL)	0.33 (0.21–0.62)	0.36 (0.20–0.70)	1.23 (0.69–2.19)	0.476
Serum IL-9 (pg/mL)	1.35 (0.83–2.10)	1.78 (1.10–3.17)	1.08 (0.96–1.22)	0.192
Serum IL-13 (pg/mL)	14.60 (10.15–24.34)	24.60 (12.91–58.53)	1.02 (1.01–1.04)	**0.005**

*Values presented as median (IQR) and as absolute number and percentage of patients; n (%). Univariate logistic regression was used with odds ratios (ORs) and 95% confidence intervals (95%CI).*

* *Above reference intervals according to age. Symptom duration n = 79 and 57. S-IL-4 n = 72 and 50, since some values were unmeasurable. S-IL-9 n = 78 and 55. S-IL-13 n = 78 and 57. N/A: not applicable. The bold values indicate the p-value below 0.05*.

**Table 4:** Adjusted variables for complicated appendicitis in 138 children with appendicitis.

	**aOR (95% CI)**	**Uncomplicated appendicitis Complicated appendicitis**		***p*-value**
Serum IgE elevated^*^	0.53 (0.24–1.18)	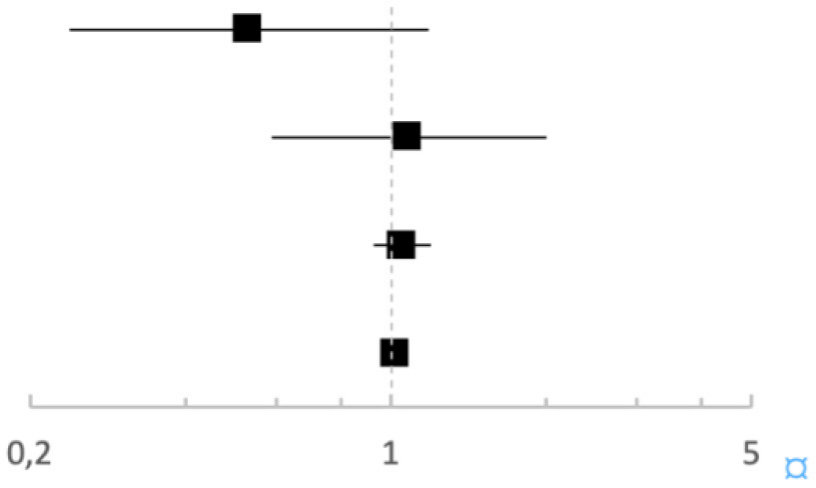		0.121
Serum IL-4 (pg/mL)	1.08 (0.59–1.99)			0.810
Serum IL-9 (pg/mL)	1.05 (0.93–1.19)			0.455
Serum IL-13 (pg/mL)	1.02 (1.01–1.04)			**0.011**

*Adjusted logistic regression was used with adjusted odds ratios (aORs) and 95% confidence intervals (95% CI).*

* *Above reference intervals according to age. Adjusted for age, symptom duration and presence of appendicolith. The bold values indicate the p-value below 0.05*.

The risk of complicated appendicitis in the crude analysis was also associated with age [OR 0.84 (95% CI 0.75–0.94), *p* = 0.003], a symptom duration of 48–96 h compared to <24 h [OR 3.27 (95% 1.22–8.71), *p* = 0.02], and the presence of an appendicolith [OR 2.86 (95% CI 1.28–6.41), *p* = 0.01] (Table 3), but significance only remained for age in the adjusted analysis (Supplementary Table 4).

## Discussion

This cohort study aimed to evaluate the association of serum concentrations of IgE, IL-4, IL-9, and IL-13 in children with the risk of complicated appendicitis. In the logistic regression, high concentrations of IL-13 were associated with an increased risk of complicated appendicitis, even when adjusting for age, symptom duration and the presence of an appendicolith–a finding that might offer novel future diagnostic possibilities. Concentrations of IgE and IL-4 and IL-9 were not significantly associated with the risk of complicated appendicitis.

Previous studies (11–14) have suggested that a person's immune response propels the inflammation toward an uncomplicated or a complicated disease course, where complicated appendicitis is associated with a T-helper (Th) 1/Th17-dependent immune response and uncomplicated appendicitis is associated with a Th2-dependent immune response. Based on this hypothesis, we conducted our previous studies on the association between (the Th2 associated) IgE-mediated allergy and a lower risk of complicated appendicitis (15, 16), which have now been continued in the present study through measurement of the IgE-levels. Previous studies from other authors have found a significant increase in IgE deposits in phlegmonous appendices compared to incidentally removed appendices, but not compared to gangrenous appendicitis and negative appendectomies (17). Furthermore, two studies have reported significant associations of eosinophilia and uncomplicated appendicitis in children (18, 19). Finally, in pregnancy (during which the pregnant woman's inflammatory response is driven toward a Th2-response), the standardized incidence ratio for perforated appendicitis is substantially lower which is then followed by a rebound effect post-partum (13).

The hypothesis that cytokines reflect different types of inflammatory responses (Th1/Th17, Th2) which could be used as biomarkers in patients with appendicitis has also been proposed. In a previous study investigating concentrations of Th2 cytokines in appendicular lavage in 41 adults, concentrations of IL-4 were significantly higher in the patients with phlegmonous appendicitis compared to those with normal appendices, but not compared to the patients with gangrenous appendicitis (20). Associations have been found between Th17-associated cytokines and complicated appendicitis, indicating that the production of these inflammatory mediators can exaggerate the immune response (14).

In the present study, children with uncomplicated appendicitis did not have significantly higher levels of IgE compared to children with complicated appendicitis. Neither were there any significant differences in the concentrations of IL-4 or IL-9 between the two groups. In contrast to our hypothesis, concentrations of the Th2-associated IL-13 were significantly elevated in the children with complicated appendicitis. This is an interesting finding, warranting future research on the diagnostic performances of IL-13, preferably in conjunct with a clinical prediction score for pediatric appendicitis (21).

There may be several reasons behind the present results. First, the lack of significant associations between serum IgE and complicated appendicitis might be due to lack of power, since not even known risk factors for complicated appendicitis such as appendicolith (22) and longer symptom duration (23) significantly affected the risk of complicated appendicitis in the adjusted analysis.

Regarding the interleukins, possibly the inflammatory answer in different types of appendicitis is not that specific as previously stated, and hence Th2-associated interleukins are not suitable as biomarkers for a lower risk of complicated appendicitis. The Th2-associated interleukins are said to act in an anti-inflammatory manner, and perhaps this is the reason for their higher levels in patients with complicated appendicitis in whom there is often a severe, systemic inflammation.

IL-13 is produced by T helper cells, NK cells, mast cells, basophils and eosinophils and is a key factor in the synthesis and maintenance of IgE production (24, 25). A recent study has also shown an association of single nucleotide polymorphisms (SNPs) of IL-13 with appendicitis, but not for severe appendicitis (26). This study highlights the association between Th2 and uncomplicated appendicitis, in contrast to the results of the present study.

A strength of the present study is that it was prospective, and that there are few previous studies that have evaluated these interleukins and their role in the inflammatory response in pediatric appendicitis. Another strength was that histopathology was used for all cases to confirm the diagnosis. We also believe that the present cohort, with a 40% rate of complicated appendicitis, is quite representative of this patient group. The main limitation of the study is the rather low number of patients. Furthermore, almost 40 patients were excluded from the original 215 and we cannot exclude that this may introduce bias to the results.

## Conclusion

High concentrations of serum IL-13 seem to be associated with an increased risk of complicated appendicitis in children, while serum total IgE, IL-4 and IL-9 do not seem to be associated with the risk of complicated appendicitis. This is incongruent with the hypothesis of a Th1/Th17-driven inflammation in this type of appendicitis and a Th2-driven inflammation in uncomplicated appendicitis.

## Data Availability Statement

The raw data supporting the conclusions of this article will be made available by the authors, without undue reservation.

## Ethics Statement

The studies involving human participants were reviewed and approved by Regionala Etikprövningsnämnden, Lund, Sweden. Written informed consent to participate in this study was provided by the participants' legal guardian/next of kin.

## Author Contributions

JG, MS, and LH designed the study and oversaw the inclusion of study participants. BR performed the laboratory analyses. JG performed the statistical analyses and drafted the manuscript. All author interpreted data, critically reviewed the manuscript, and approved the final version.

## Funding

This study was funded by Region Skåne (JG) and Bengt Ihre Research Fellowship (MS).

## Conflict of Interest

The authors declare that the research was conducted in the absence of any commercial or financial relationships that could be construed as a potential conflict of interest.

## Publisher's Note

All claims expressed in this article are solely those of the authors and do not necessarily represent those of their affiliated organizations, or those of the publisher, the editors and the reviewers. Any product that may be evaluated in this article, or claim that may be made by its manufacturer, is not guaranteed or endorsed by the publisher.
