# Supplementary material for: An Evaluation of Serum IgE and Th2-Associated Interleukins in Children With Uncomplicated and Complicated Appendicitis
Source: Front Pediatr. 2022 May 2;10:884138. doi: 10.3389/fped.2022.884138 (PMC9108389; doi:10.3389/fped.2022.884138)
Supplement: Supplementary file 1 [file Data_Sheet_1.pdf]

Supplementary Tables and Figures

Supplementary Table 1. The age specific reference intervals of total serum IgE according to the Department of Clinical Immunology and Transfusion medicine, Region Skåne.

Age (years)	Reference interval (ng/mL)
> 1	< 31.2
> 2	< 55.2
> 3	< 76.8
> 4	< 96
> 5	< 115.2
> 6	< 134.4
> 7	< 151.2
> 8	< 170.4
> 9	< 187.2
> 10	< 204

Supplementary Table 2. Demographics and final diagnoses in 178 children with suspected appendicitis

	No appendicitis<br>n = 40	Appendicitis<br>n = 138	p-value
Age (years)	11 (9-12.75)	10 (8-12)	0.481
Sex (male)	16 (40)	87 (63)	**0.009**
Allergy	3 (8)	20 (15)	0.246
Symptom duration<br>0-24 h<br>24-48 h<br>48-96 h<br>>96 h	 14 (35)<br>13 (33)<br>9 (23)<br>4 (10)	 60 (43)<br>50 (36)<br>24 (17)<br>2 (1)	 0.051
Season<br>Spring<br>Summer<br>Autumn<br>Winter	 13 (33%)<br>11 (28%)<br>6 (15%)<br>10 (25%)	 33 (24%)<br>29 (21%)<br>32 (23%)<br>44 (32%)	 0.405
Final diagnoses	Non-specified abdominal pain (26), Mesenterial lymphadenitis (3), Ovulation (2),<br>Ileitis (1), Ovarian torsion (1), Pyelonephritis (1), Meckel's diverticulum (1), Inguinal hernia (1), Constipation (1), Upper respiratory tract infection (1), Gastroenteritis (1), Abdominal tumor (1)	Phlegmonous (80)<br>Gangrenous (23)<br>Perforated (31)<br>Abscess (4)	

Values presented as median (IQR) and as absolute number and percentage of patients; n(%). Group differences were assessed through Mann-Whitney U test for continuous data and with Chi-squared test for categorical data. Symptom duration data missing for 2 patients with appendicitis, n = 40 and 136.

Supplementary Table 3. Levels of interleukins and IgE in 178 children with suspected appendicitis

	**No appendicitis n = 40**	**Uncomplicated appendicitis n = 80**	**Complicated appendicitis n = 58**	**p-value**
**Serum IgE** (pg/mL)	121.6 (83.4-258.4)	160.8 (72.6-530.4)	127.2 (64.0-404.2)	0.326
**Serum IL-4** (ng/mL)	0.4 (0.2-0.8)	0.3 (0.2-0.6)	0.4 (0.2-0.7)	0.852
**Serum IL-9** (pg/mL)	1.9 (1.0-2.7)	1.4 (0.8-2.1)	1.8 (1.1-3.2)	0.088
**Serum IL-13**(pg/mL)	11.3 (8.4-20.2)	14.6 (10.2-24.3)	24.6 (12.9-58.5)	**0.001***

Values presented as median (IQR), group differences assessed through Kruskal-Wallis test.
S-IL-4 n = 38, 72 and 50, since some values were unmeasurable; S-IL-9 n = 40, 78 and 55; S-IL-13 n = 38,78 and 57
*Indicates significant differences between no appendicitis and complicated, and between uncomplicated and complicated appendicitis

Supplementary Table 4. Adjusted variables for complicated appendicitis in 138 children with appendicitis

	**aOR (95% CI)**	**p-value**
**Age**	0.83 (0.73-0.95)	**0.005**
**Symptom duration**<br>0-24 h<br>24-48 h<br>48-96 h<br>>96 h	 Ref<br>2.43 (1.02-5.82)<br>2.57 (0.86-7.67)<br>N/A	 Ref<br>**0.046**<br>0.090<br>N/A
**Appendicolith**	2.05 (0.84-4.95)	0.113
**Serum IgE elevated***	0.53 (0.23-1.18)	0.121
	**aOR (95% CI)**	**p-value**
**Age**	0.84 (0.73-0.96)	**0.012**
**Symptom duration**<br>0-24 h<br>24-48 h<br>48-96 h<br>>96 h	 Ref<br>2.14 (0.84-5.48)<br>2.41 (0.78-7.51)<br>N/A	 Ref<br>0.112<br>0.128<br>N/A
**Appendicolith**	1.89 (0.72-4.94)	0.195
**Serum IL-4**	1.08 (0.59-1.99)	0.810
	**aOR (95% CI)**	**p-value**
**Age**	0.83 (0.73-0.95)	**0.007**
**Symptom duration**<br>0-24 h<br>24-48 h<br>48-96 h<br>>96 h	 Ref<br>2.16 (0.90-5.15)<br>2.44 (0.80-7.38)<br>N/A	 Ref<br>0.083<br>0.116<br>N/A
**Appendicolith**	1.77 (0.71-4.40)	0.222
**Serum IL-9**	1.05 (0.93-1.19)	0.455
	**aOR (95% CI)**	**p-value**
**Age**	0.86 (0.76-0.99)	**0.029**
**Symptom duration**<br>0-24 h<br>24-48 h<br>48-96 h<br>>96 h	 Ref<br>2.43 (0.99-5.96)<br>2.78 (0.89-8.72)<br>N/A	 Ref<br>0.052<br>0.080<br>N/A
**Appendicolith**	1.83 (0.73-4.63)	0.200
**Serum IL-13**	1.02 (1.01-1.04)	**0.011**

Adjusted logistic regression was used with adjusted odds ratios (aORs) and 95% confidence intervals (95% CI).
*Above reference intervals according to age. Adjusted for age, symptom duration and presence of appendicolith.

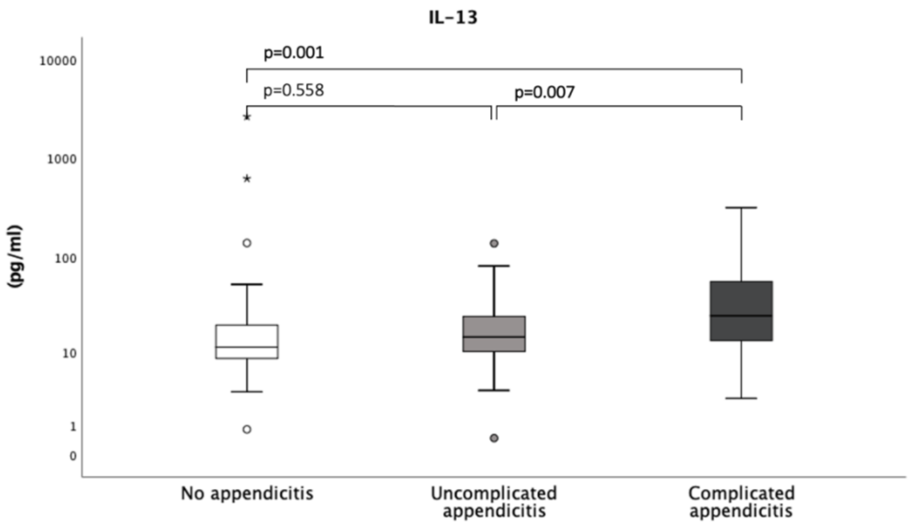

Supplementary Figure 1. Box plots of Interleukin (IL)-13 concentrations according to final diagnosis.
